# Landscape Heterogeneity and Changing Bioclimatic Parameters Link to Potential Expansion of *Gazalina chrysolopha* (Lepidoptera; Notodontidae) in Nepal: A Possible Bioagent for Seasonal Hyperacute Panuveitis (SHAPU) in South Asia

**DOI:** 10.1002/ece3.72325

**Published:** 2025-10-10

**Authors:** Daya Ram Bhusal, Ranju Kharel (Sitaula), Bimal Raj Shrestha, Suraj Baral, Bhawana Pandey, Pratikshya Pathak, Bhupendra Kumar, Pratap Karki, Sagun Narayan Joshi, Ananda Kumar Sharma, Madan Prasad Upadhya

**Affiliations:** ^1^ Central Department of Zoology Tribhuvan University Kathmandu Nepal; ^2^ B.P. Koirala Lions Centre for Ophthalmic Studies, Institute of Medicine Tribhuvan University Kathmandu Nepal; ^3^ Biodiversity Research and Conservation Society Kathmandu Nepal; ^4^ Amrit Campus, Department of Zoology Tribhuvan University Kathmandu Nepal; ^5^ Leibniz Institute for the Analysis of Biodiversity Change, Museum Koenig Bonn Bonn Germany; ^6^ Department of Zoology Banaras Hindu University Varanasi India; ^7^ Department of Ophthalmology B.P. Eye Foundation Bhaktapur Nepal

**Keywords:** distribution, ecological niche modeling, eye disease, moth, SHAPU

## Abstract

Seasonal hyperacute panuveitis (SHAPU) is an eye disease primarily reported in Nepal, especially in mid‐hill regions of central and western parts. Several clinical and entomological studies have identified *Gazalina chrysolopha* as the possible causative agent for this disease. This study aimed to identify how the landscape structure and bioclimatic factors are shaping its distribution in the Nepal Himalayas. We performed extensive surveys in various regions of Nepal, focusing on previous and current SHAPU‐reported sites in the country. We performed extensive observations in all possible habitats across the sites, and records were made with GPS points indicating the presence of adult moths. The surveys were conducted from March to October over two consecutive years: March 2023 to October 2024. We supplied the presence GPS points with freely available bioclimatic, topographic, vegetation, and landscape‐related variables to model habitat suitability and to predict both current and future distributions of *G. chrysolopha* using the Maxent algorithm. Our analysis indicated that landscape heterogeneity combined with bioclimatic parameters is a major determinant of the spatial distribution of this species of moth in Nepal. Our study found that the moth exhibits a higher population presence in the mid‐mountains of Nepal. Our model predicted that the eastern part of Nepal has a higher probability of habitat suitability for this species under scenarios of climate and rapid landscape change. The relative importance of different bioclimatic variables and local landscape features, such as forest proportion, forest‐urbanization edges, and the presence of specific microhabitats, has been identified as affecting its distribution. Ongoing changes in climate and landscape features are likely to affect the future distribution of this moth species in Nepal. All climate and land cover change scenarios suggest that the suitable habitat for the moth will more than double its current range.

## Graphical abstract

Seasonal hyperacute panuveitis (SHAPU) is an eye disease in Nepal. Several clinical and entomological studies have identified *Gazalina chrysolopha* as the possible causative moth for this disease.

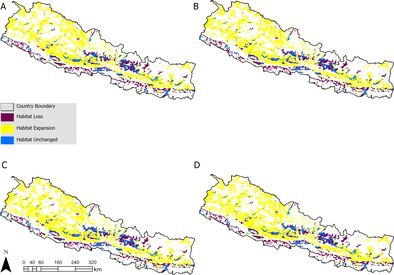

## 1 Introduction

Lepidopterans, which include butterflies and moths, are among the most widely distributed groups of insects (Mally et al. 2024; Stork 2018). They have been extensively studied to gain insights into various ecological and evolutionary processes, as this group exhibits a significant response to climate change (Hill et al. 2017; Wilson and Maclean 2011). As global temperatures rise, insects show a high sensitivity to changes in temperature and climatic conditions (John et al. 2024; Hill et al. 2021; Parmesan and Yohe 2003). A notable response to these changes in distribution patterns is that many species are shifting their ranges towards cooler regions or higher elevations (Choi et al. 2022; Lehmann et al. 2020). As a consequence, it is suggested that extreme climatic events can significantly affect the taxonomic, functional, and phylogenetic diversity of insects, altering community dynamics and leading to changes in species composition and species extinction (Hill et al. 2021; Ohlberger 2013; Thomas 2005). On the other hand, landscape features such as topography, vegetation dynamics, habitat mosaics, microclimatic characteristics, and land use patterns significantly influence species distribution (Coelho et al. 2023; Uhl et al. 2021) in various insect groups, including butterflies and moths. Additionally, the availability of habitats, competition among species for resources, and optimal environmental conditions play a significant role in the abundance and distribution of lepidopterans across landscape scales. This relationship is notably affected by various factors, including spatial scales, local weather, and regional climate characteristics. Furthermore, land use conditions play a vital role in shaping these dynamics (Bottero et al. 2023; Campos et al. 2013). Under rapid land‐use change and climate change, landscape heterogeneity along elevation gradients has become a significant environmental filter for the ecological assembly of many insect communities (Bhusal and Khanal 2008; Ghimire et al. 2023; Ibalim et al. 2024; Shrestha et al. 2024; Timberlake et al. 2024).

The diversity and abundance of insects vary by the landscape configuration. These changes have notable effects on multiple aspects of insect life history, such as fertility, feeding patterns, survival rates, population dynamics, and dispersal, including moth groups (Fuentes‐Montemayor et al. 2012; John et al. 2024; Ricketts et al. 2001). Some studies that examine the relationships between landscape structure and moth abundance in fragmented habitats have shown considerable variation and highlighted the risks of environmental changes to survival and distribution (John et al. 2024; Płóciennik et al. 2023; Summerville and Crist 2008). Additionally, moths are also influenced by other ecological factors, including microhabitat types, local‐landscape vegetation dynamics, and microclimatic types (Bhattacharjee et al. 2017; Highland et al. 2013; Tyler 2020). Therefore, understanding this relationship is crucial for predicting how global environmental changes will influence the habitat suitability of moths across space and time (Highland et al. 2013).

Moths are highly abundant lepidopterans that are found in all trophic levels and are specialized for terrestrial environments (Płóciennik et al. 2023). They play an important role in maintaining vegetation dynamics and fulfill several critical ecological functions in different ecosystems (Hahn and Brühl 2016; New 2023; Ratnamsingham and Decaëns 2022; Toko et al. 2023; Wagner et al. 2021), such as pollinators for many natural and cultivated plants (Finch et al. 2021; Macgregor et al. 2015; Ribas‐Marquès et al. 2022; Singh et al. 2022), contributing to herbivory, and acting as a vital food source for many predatory animals, including birds and mammals (Ancillotto et al. 2023; Augusto et al. 2025; Kolkert et al. 2020). Therefore, they help to maintain ecosystem balance, support plant reproduction, and provide ecosystem services (Dangles and Casas 2019).

Many moth species are sensitive to temperature and adversely affected by environmental changes (Hill et al. 2021; Keret et al. 2020). These impacts often result in life history strategies, distribution patterns, and phenology (Hickinbotham et al. 2024). On the other hand, ecological interactions, particularly those between plants and moths, are greatly influenced by bioclimatic changes and habitat loss (Kühne et al. 2022; Brosi et al. 2008; Rösch et al. 2013). Climate change influences the availability of host plants, which in turn affects the population (Kadlec et al. 2009) and composition of moth species (Hill et al. 2021; Mangels et al. 2017; Navarro‐Cano et al. 2015). They also serve as significant potential bioindicators (Summerville et al. 2004) as they exhibit a complex array of geographical responses to climate change, with some species facing local extinctions while others have shifted their geographical ranges in response to global changes (Kadlec et al. 2009; Hällfors et al. 2024; Hickling et al. 2006).

The distribution, ecological modeling, and responses of moths to climate change remain largely underexplored. Additionally, the medical significance of various moth species is also poorly studied. Three species of *G. chrysolopha, G. apsara*, and G. transversa have been identified in Nepal (Khanal and Shrestha 2022). In the context of the Hindu‐Kush Himalayan regions, *G. chrysolopha* has been reported in several other South Asian countries, particularly within the Himalayan belt (Uniyal et al. 2016; Bhattacharyya et al. 2019; Shah et al. 2017; Tamang et al. 2023). Taxonomically, this species has been thoroughly studied in this region (Dewan et al. 2022; Kaleka et al. 2024; Khanal and Shrestha 2022). The localities for *G. chrysolopha* have been noted in northern Pakistan, including Kashmir (Rahman and Chaudhry 1992), as well as in certain areas of China (Wu and Fang 2002). Notably, *G. chrysolopha* has been linked with seasonal hyperacute panuveities (SHAPU) in Nepal, highlighting the need for further research into its ecological and health impacts (Gurung et al. 2021; Kharel et al. 2020; Upadhyay et al. 2019, 2021). Previous studies have shown that *G. chrysolopha* was primarily found in the mid‐ecological zones of Nepal, particularly in the Pokhara and Kathmandu valleys and their surrounding districts (Gurung et al. 2021; Khanal and Shrestha 2022). However, local observations indicate a significant shift in its distribution, as it is now spreading towards the highlands and eastern regions of Nepal. This development calls for further research into the causes behind the expanding range of this moth, as well as the potential health risks it poses to local communities. Specifically, the increasing distribution of *G. chrysolopha*, along with its interactions with landscape characteristics and favorable bioclimatic factors at both local and broader landscape scales, has yet to be thoroughly explored. The caterpillar of *G. chrysolopha* is known to be a significant defoliator of *Alnus nepalensis* in Nepal (Khanal and Shrestha 2022), as well as in India and Bhutan (Srivastava and Mukhopadhyay 2006). In northern Pakistan, it poses a considerable threat to *Quercus dilata* (Rahman and Chaudhry 1992). Furthermore, Srivastava and Mukhopadhyay (2006) noted that the larvae of *G. chrysolopha* are also major defoliators of A. nepalensis in Sikkim, India.

Increasing cases of SHAPU have concurrently been reported from the eastern regions of Nepal, where *G. chrysolopha* is spreading rapidly. However, the epidemiological relationship between moth species and SHAPU remains poorly understood. In this context, it is essential to monitor and understand the factors influencing the distribution of this moth in districts of Nepal affected by SHAPU, especially considering the potential impacts of climate change and rapid alterations to bioclimatic parameters and landscape features. Therefore, documenting the diverse ecological aspects and habitat preferences of this species is crucial for developing an ecological framework that can inform preventive strategies against SHAPU in Nepal. Exploring these relationships could provide valuable insights for public health strategies and can help in the development of an effective integrated medical protocol for the prevention of SHAPU in Nepal.

The main objective of this study is to analyze how the distribution of *G. chrysolopha* changes over the years under various emission scenarios. We aim to determine whether these changes are linked to climate factors and landscape features. Specifically, we aim to identify key bioclimatic variables and aspects of landscape diversity, including forest dynamics, geographical features, urban influence, and habitat mosaics, that significantly affect the distribution of *G. chrysolopha* in Nepal.

## 2 Materials and Methods

### 2.1 Collection of *G. chrysolopha* Presence Data

We conducted surveys across Nepal, focusing specifically on sites that have previously reported and are currently reporting SHAPU. This is a rare and severe eye disease that mostly affects children and leads to rapid vision loss, often resulting in blindness. Its symptoms include sudden, intense inflammation within the eye (panuveitis). We identified 419 presence points across 44 districts (sites) of *G. chrysolopha*. At each site, we thoroughly examined all potential habitats and collected GPS coordinates indicating the presence of adult *G. chrysolopha*. The surveys were conducted from March to October over two consecutive years: March 2023 to October 2024. This timeframe was selected based on the higher activation periods of the moth (Manandhar et al. 2018; Khanal and Shrestha 2022; Sapkota et al. 2022). Fieldwork was conducted between 6:00 PM and 11:00 PM under suitable weather conditions, with no rainfall. During the data collection, we used long waterproof jackets, masks, gloves, and eyeglasses for safety purposes. The final dataset comprises 419 presence points of the moth (see File S1). We subjected these presence points to data thinning using the SDM Toolbox 2.0 add‐in (Brown et al. 2017) in ArcGIS Pro to remove points closer than 1 km, thus mitigating geographic bias and spatial autocorrelation in the data (Boria et al. 2014).

### 2.2 Environmental Data

We utilized freely available environmental datasets to assess the suitability of the species, including bioclimatic, topographic, and landscape features. These features encompass habitat‐related variables, topographic, and disturbance‐related factors. For bioclimatic variables, we downloaded 19 variables from WorldCLIM 2.0 (Fick and Hijmans 2017), which are widely used to evaluate the potential responses of species to climate change and to assess climatic suitability under current and future climate scenarios.

We also obtained the latest MODIS land‐cover dataset to identify built‐up areas as urban land cover. We then calculated the Euclidean distance from urban land cover to use as a disturbance‐related variable. Additionally, we extracted all forested pixels from the same dataset to calculate the proportion of forest within a circular window of three kilometers. Furthermore, we used the MODIS data to calculate the spatial heterogeneity of available land cover using Shannon's Diversity Index (Shannon and Weaver 1949). The proportion of forest and spatial heterogeneity were used as habitat‐related variables in the modeling procedure. The Digital Elevation Model (DEM) of the study area, also available from WorldCLIM 2.0, was used to calculate topographic variables. Specifically, the position of geography relative to geographic north (Northness) and east (Eastness) was computed. We performed an initial candidate set that included 24 environmental variables (Table 1), followed by applying the Variance Inflation Factor (VIF < 5) test in R, which was narrowed down to 11 uncorrelated variables.

**TABLE 1:** Bioclimatic, landscape, and topographic variables applied in the modeling.

Variables used	Code used in the text	Unit
*Bioclimatic variables*		
Annual mean temperature	bio1	°C
Mean diurnal range (mean monthly (max temp—min temp))	bio2	°C
Isothermality (BIO2/BIO7) (×100)	bio3	%
Temperature seasonality (SD ×100)	bio4	%
Max temperature of warmest month	bio5	°C
Min temperature of coldest month	bio6	°C
Temperature annual range (BIO5‐BIO6)	bio7	°C
Mean temperature of wettest quarter	bio8	°C
Mean temperature of driest quarter	bio9	°C
Mean temperature of warmest quarter	bio10	°C
Mean temperature of coldest quarter	bio11	°C
Annual precipitation	bio12	mm
Precipitation of wettest month	bio13	mm
Precipitation of driest month	bio14	mm
Precipitation seasonality (coefficient of variation)	bio15	%
Precipitation of wettest quarter	bio16	mm
Precipitation of driest quarter	bio17	mm
Precipitation of warmest quarter	bio18	mm
Precipitation of coldest quarter	bio19	mm
*Landscape variables*		
Proportion of forest = proportion of forest in study sites	Prop_forest	%
Spatial heterogeneity: spatial heterogeneity of landscape	SH_IN	unitless
Distance to build‐up areas	Dist_urban	(m)
Topographic variables		
Northness: Northern aspect of landscape	Northness	degrees
Eastness: southern aspect of landscape	Eastness	degrees

### 2.3 Current and Future Habitat Suitability Analysis

The Maximum Entropy Model (Maxent) (Phillips et al. 2006) was employed to predict the habitat suitability for the species. A final set of variables was used to train the habitat suitability model. Maxent is well‐suited for presence‐only data, and small sample size (Hernandez et al. 2006) offers superior predictive power compared to other distribution models (Elith et al. 2006; Wisz et al. 2008) and can handle the non‐linear relationships between predictors and response variables (Elith et al. 2006). We ran 25 replicates for each model, following Bradsworth et al. (2017). Each replicate used 60% of the available presence points and 5000 background points, which were obtained from a Gaussian Kernel‐based bias file using the *dismo* package in R (Hijmans et al. 2024). The remaining presence locations were used for model validation. We evaluated the model's performance using true skill statistics (TSS) (Allouche et al. 2006) and the area under the curve (AUC) of the receiver operating characteristic (ROC) curve (Fielding and Bell 1997). All models were run in R (R Core Team 2021) using the sdm 2.0 package (Naimi and Araújo 2016). To translate the continuous suitability values into presence and absence, we used the threshold that maximizes TSS (Liu et al. 2016). Before running the final model, we adjusted the regularization multiplier and feature combinations in the R environment using ENMeval 2.0 (Kass et al. 2021). We tested a range of linear, quadratic, and hinge features with regularization multipliers from 0.5 to 5. The optimal model was determined based on Delta AICc, with a ∆AICc value of zero indicating the best model (Burnham and Anderson 1998).

The correlation tests available in the sdm package (Naimi and Araújo 2016) were used to assess the importance of each variable in the models. This test involved randomly permuting the variable of interest and calculating the correlation between the predicted values and the permuted values. A higher correlation between these values indicates lower variable importance, as permutation affects the prediction (Thuiller et al. 2009; Naimi and Araújo 2016). Variable importance was quantified as “1−correlation” (Thuiller et al. 2009), with the measure derived from the mean importance across all models. Finally, using the Digital Elevation Model (DEM), we determined the elevational distribution of the moth. We projected the current model into future climatic conditions using the MIROC6 global circulation model (GCM), a widely used global climate model. We assessed potential habitat for the species under near‐future (2021–2040) and mid‐future (2041–2060) scenarios, considering two emission scenarios: the intermediate (SSPs 2‐4.5) and the high (SSPs 5‐8.5) emissions scenarios. Future bioclimatic variables for these periods and scenarios were downloaded from WorldCLIM version 2.1 (Fick and Hijmans 2017). For habitat and disturbance‐related variables, we used the Global Land Use and Land Cover Change product derived from MODIS land cover data. We accessed data for two emission scenarios: A1B (representing the intermediate scenario) and A2 (representing the high emission scenario), focusing on the year 2060 following Baral et al. (2023). Topographic variables were kept constant, as they do not change under future climate scenarios.

## 3 Results

### 3.1 Model Performance and Variable Importance

**FIGURE 1:**
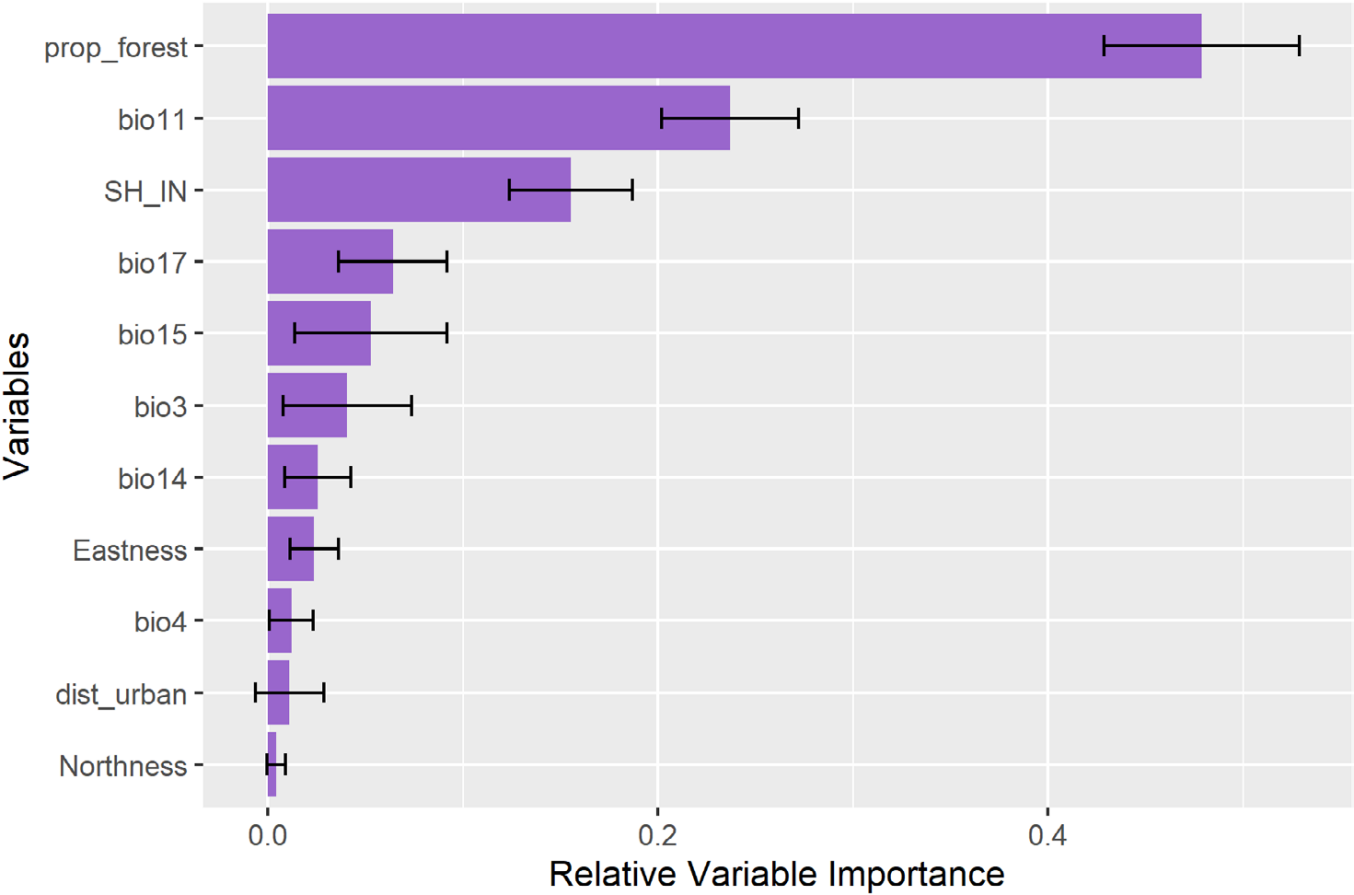
The relative importance of bioclimatic and landscape variables (Table 1).

The evaluation metrics, AUC (0.81 ± 0.03) and TSS (0.53 ± 0.06), indicated that the model has very good discriminative ability. The variable importance analysis revealed that habitat‐related factors, such as forest proportion, habitat diversity (Shannon index), and Mean Temperature of the Warmest Quarter, were the primary predictors that have a significant impact on the moth's distribution (Figure 1). Similarly, Precipitation Seasonality and Precipitation of Driest Quarter were the next most important bioclimatic variables influencing the distribution of this moth species. Conversely, some bioclimatic variables, like Isothermality, Temperature Seasonality, Precipitation of Driest Month, and environmental variable Eastness, were the least influential in limiting the distribution of *G. chrysolopha* in our study. Additionally, we found that ecological factors, like distance to urban areas and northern aspect (Northness), did not play a significant role in the distribution of this species in our study areas.

**FIGURE 2:**
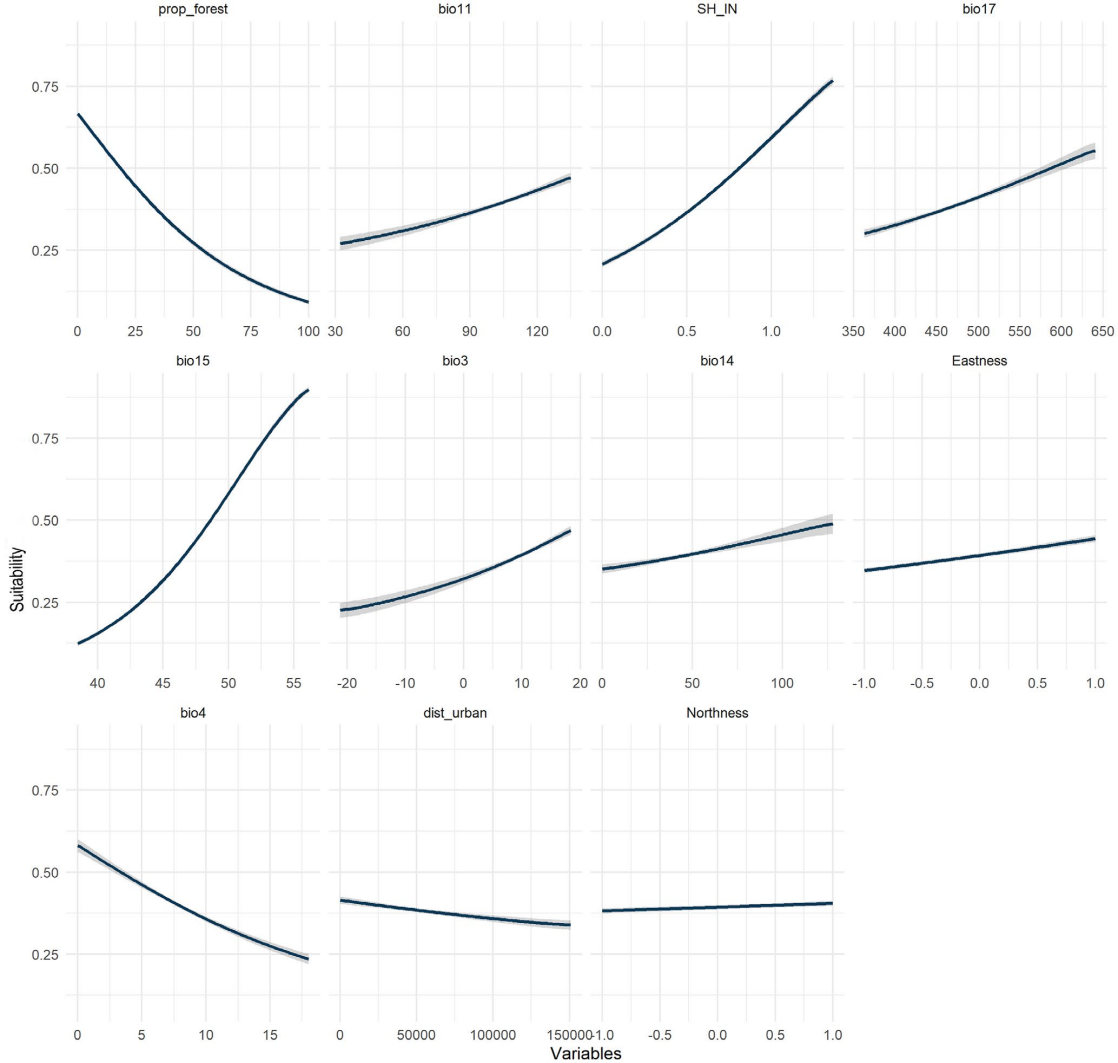
Response curve for variables (Table 1) used to model the habitat suitability of *Gazalina chrysolopha* in Nepal as predicted by the maximum entropy (maxent) model.

The response curve for the key variables mentioned above was analyzed (Figure 2), and it was observed that habitat suitability increased with habitat heterogeneity (Shannon index), along the Eastness, Precipitation of Driest Month, Isothermality, and Precipitation of Driest Quarter. Conversely, suitability decreased with an increase in forest proportion and Temperature Seasonality. There was no specific relationship with Northness or distance to urban areas.

### 3.2 Habitat Suitability of *G. chrysolopha*

**FIGURE 3:**
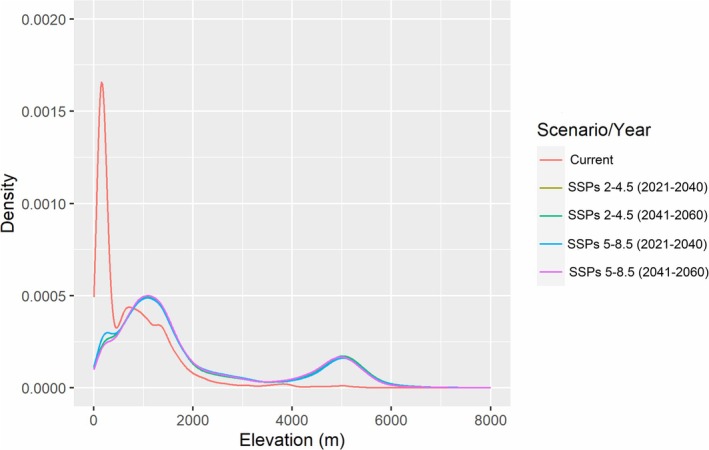
The density plot of the suitable elevation of *Gazalina chrysolopha* in all the emission scenarios—SSPs 2‐4.5 and 5‐8.5.

**FIGURE 4:**
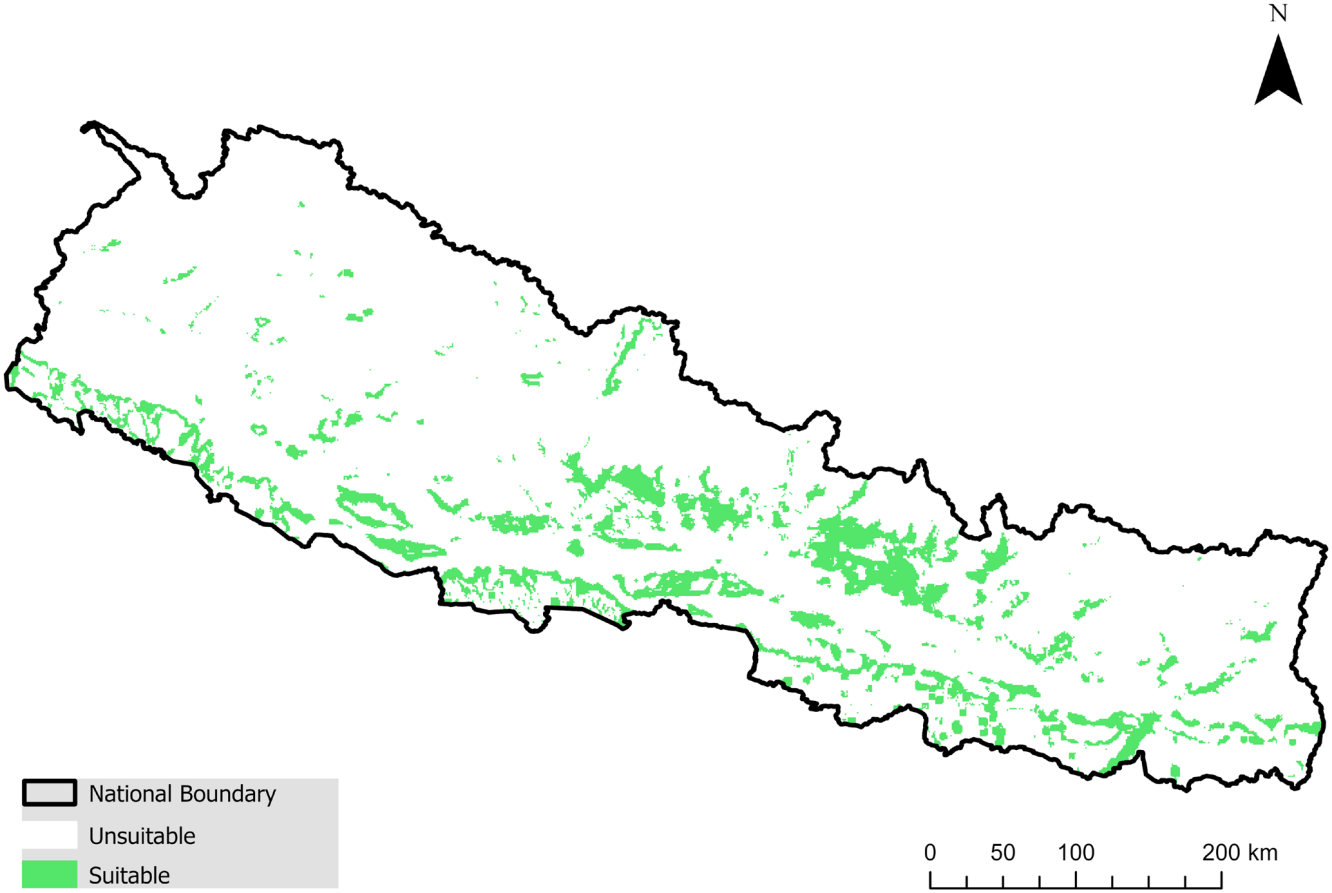
Current habitat distribution of *Gazalina chrysolopha* in Nepal as predicted by the Maxent model in this study based on presence background data.

The present study revealed that the mid‐mountains of Nepal, ranging from 1500 to 2000 m above sea level (asl), provide suitable habitats for *G. chrysolopha* (Figure 3). Whereas the lowest habitat suitability was observed in the lowland areas as well as the high mountains of Nepal with more alpine and subalpine climatic features proximal to the Himalayas (Figure 4). In our analysis, the density plot of the suitable habitat showed that the lowland of Nepal currently has the maximum suitable habitat. However, in all scenarios, the most preferred habitat will shift towards the mid and high mountains of Nepal.

**FIGURE 5:**
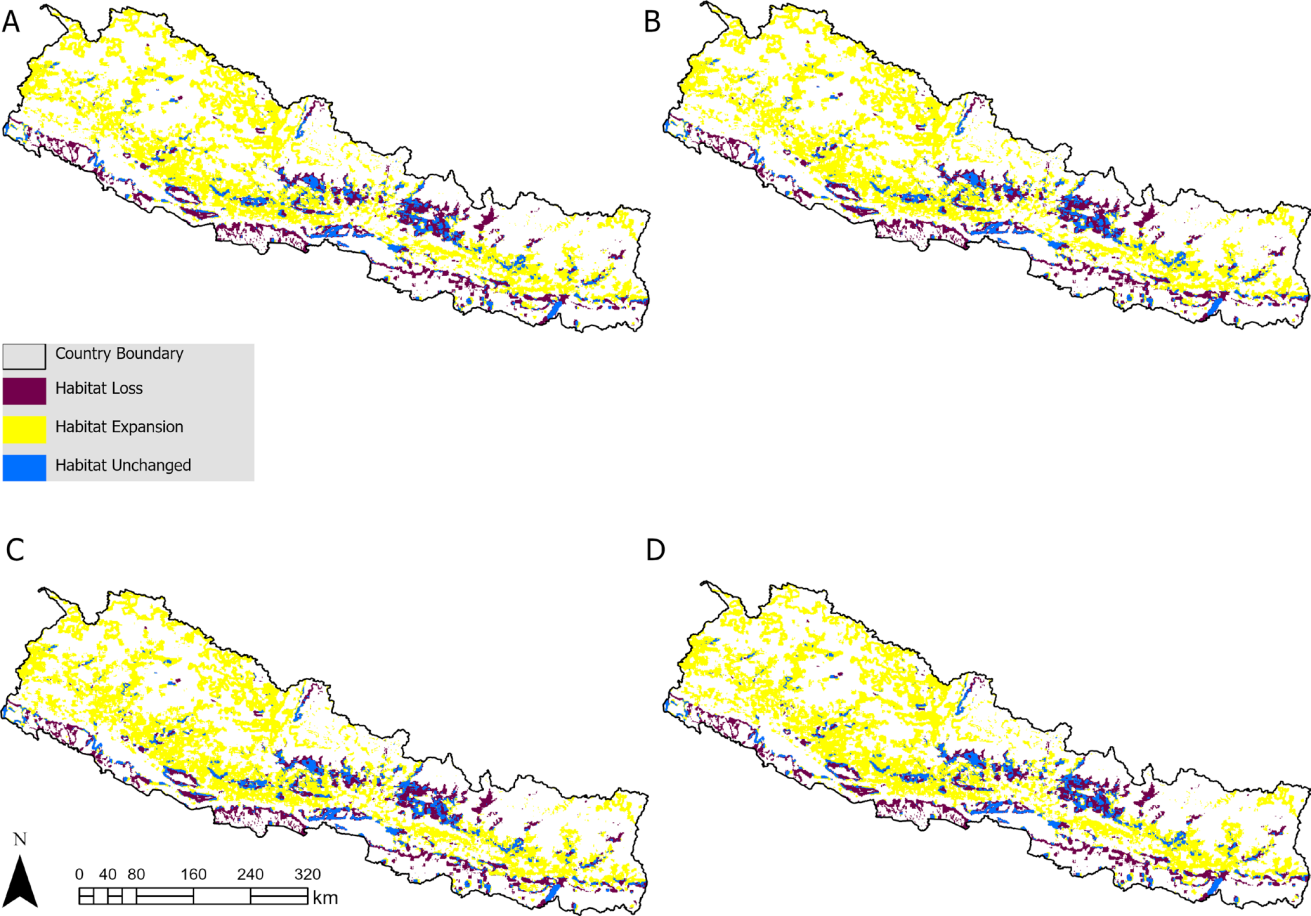
Absolute change in potential distribution of *Gazalina chrysolopha* in Nepal under intermediate (A, B) and high (C, D) greenhouse gas emissions scenarios (shared socioeconomic pathways) for 2021–2040 (A, C) and 2041–2060 (B, D).

All climate and land cover change scenarios indicate an expansion of more than twice the currently suitable habitat for the species (Figure 5). The greatest expansion (~128%) is projected to occur under the high‐emission scenario in the near future, followed by an expansion of approximately 127% under the high‐emission scenario in the mid‐future (Table 2). We found that most of the expansions will occur in the mid‐mountain region (Figure 4). In contrast, the Terai area is expected to experience the most significant contraction of suitable habitat in all future scenarios. Approximately half of the currently suitable habitat is anticipated to remain intact (Table 2).

**TABLE 2:** Absolute change in suitable areas analyzed under Shared Socio‐economic Pathways (SSPs 2–4.5 and 5–8.5) emission scenarios for 2021–2040 and 2041–2060 with respect to the current potential distribution of *Gazalina chrysolopha*.

Scenario	Years	No change (km^2^)	Expansion (km^2^)	Loss (km^2^)
Intermediate SSPs (2–4.5)	2021–2040	9524	48,590	13,436
	2041–2060	9549	48,707	13,411
High SSPs (5–8.5)	2021–2040	10,090	52,574	12,870
	2041–2060	9624	52,109	13,348

## 4 Discussion

The rapid habitat expansion of this climate‐sensitive moth is likely to reflect broader ecological changes, which may be driven by bioclimatic parameters, land use patterns, and host plant dynamics (Eickermann et al. 2023; Rubenstein et al. 2023; Harvey et al. 2020; Sanyal et al. 2013). We analyzed the distribution of *G. chrysolopha* in Nepal, taking into account current and future climatic and landscape conditions. Our modeling revealed that this moth species is predominantly found in the mid‐mountain regions, including districts around the west (Kaski, Parbat, Myagdi, Gorkha, Lamjung, and Baglung) and central (Kathmandu valleys, Kavreplanchok, Dhading, and Nuwakot) Nepal, particularly in forested urban interfaces with a high abundance of *Alnus nepalensis* trees. The recent expansion of this species towards eastern parts of Nepal and its significant abundance present challenges in understanding how climate change and increasing landscape variability affect its distribution patterns, both in its native sites (Pokhara Valley and its surrounding areas) and in the newly expanding areas towards eastern Nepal.

Our results show that the distribution of *G. chrysolopha* is closely associated with landscape spatial heterogeneity, particularly as this species favors the microhabitats of urban forest interfaces well dominated by *A. nepalensis*. This habitat offers favorable microclimates, including suitable temperatures and host plants like *A. nepalensis* , which are crucial for caterpillar development. The moth's preference for these areas underscores a significant ecological relationship with the distribution of this tree species. *A. nepalensis* is abundant in the mid and eastern regions of Nepal, where the distribution of the moth is most prominent (Rana et al. 2018). In contrast, the edge of urban forests is marked by increased artificial lighting (Brehm et al. 2021; Hakbong et al. 2021; van Langevelde et al. 2011), which may attract this moth species. This attraction poses a higher risk of causing SHAPU in this area. This is likely related to the availability of suitable food plants and microhabitats that are essential for moth metamorphosis (Lintott et al. 2014). In our analysis, we found that the proportion of forest cover had a negative correlation with the distribution of *G. chrysolopha*. This may be because the moth favors specific host plants, such as *A. nepalensis*, as well as particular microhabitats and microclimates, rather than areas with a more general forest cover that includes a wider diversity of vegetation (Gurung et al. 2021; Rahman and Chaudhry 1992). Our model suggests that the availability of *A. nepalensis* , combined with favorable climatic conditions in the mid‐mountains of Nepal, is the key geographic factor influencing the distribution of this species. The presence of suitable sites for larval and pupal development, as well as overwintering sites (microhabitat under the host tree) and the prevalence of host plants, plays a significant role in the abundance and distribution of *G. chrysolopha* in these specific locations.

In other aspects, it is possibly associated with suitable bioclimatic variables, such as Precipitation Seasonality, which influence moisture and humidity levels, which in turn optimize the distribution and population stability of *G. chrysolopha* towards the eastern region (Wang et al. 2023). Therefore, the rapid shift of *G. chrysolopha* towards eastern Nepal may be driven by the increased availability of its host plants (Rana et al. 2018), including *A. nepalensis* , as well as favorable bioclimatic variables like increased precipitation and rich vegetation dynamics towards the region (Highland et al. 2013).

Moreover, behavioral plasticity is key for ectothermic organisms like moths, which must adapt to changing environmental conditions. This adaptability is vital for their resilience in the face of rapid distribution changes. As environmental conditions shift, *G. chrysolopha* may adjust its life cycle strategies, such as overwintering in earlier stages, to cope with climate changes, potentially mitigating the phenological mismatch with its host plant (van Dis et al. 2023).

By monitoring these trends, we can gain a better understanding of how climate and landscape changes impact *G. chrysolopha* in light of environmental shifts. Climate change is expected to accelerate these distribution shifts, as variations in temperature and moisture will create more favorable conditions for the moth. Changes in moisture and precipitation patterns are likely to influence population dynamics, particularly reproductive success. The observed outbreaks of *G. chrysolopha* from September to October appear to be closely related to the average temperatures during these months. This suggests a strategic adaptation in their life cycle that allows the species to thrive despite fluctuations in environmental conditions (Banko et al. 2022). This study emphasizes the importance of landscape structure, particularly in the mid‐mountains of Nepal, in influencing the rapid distribution of *G. chrysolopha*.

Future studies should sample a broader range of landscapes to capture diverse ecological conditions. A more extensive spatial coverage and systematic sampling of presence and absence may better capture the environmental and geographical diversity of Nepal, providing a stronger predictive model for the habitat suitability study of this species. By identifying hotspot regions for SHAPU, future research can concentrate on targeted data collection related to moth populations, SHAPU disease, local microclimates, and the availability of host plants.

Our predictive models suggest that rapidly changing temperature patterns, related bioclimatic factors, and landscape features are likely to cause the expansion of *G. chrysolopha* into various ecological regions of Nepal. In the future, we can improve the accuracy of our predictions and gain deeper insights into the factors affecting the distribution of *G. chrysolopha* and its relationship with SHAPU by employing advanced modeling methods that take into account bioclimatic features and landscape characteristics at a different scale. This comprehensive approach will be essential for developing integrated preventive models and early warning systems to address SHAPU at the community level.

## Author Contributions

**Daya Ram Bhusal:** conceptualization (lead), data curation (equal), formal analysis (lead), funding acquisition (equal), investigation (lead), methodology (equal), project administration (equal), resources (equal), software (supporting), supervision (lead), validation (lead), visualization (lead), writing – original draft (lead), writing – review and editing (lead). **Ranju Kharel (Sitaula):** conceptualization (equal), data curation (equal), funding acquisition (lead), project administration (lead), resources (equal), software (supporting), supervision (equal), visualization (equal), writing – original draft (supporting), writing – review and editing (equal). **Bimal Raj Shrestha:** data curation (equal), formal analysis (equal), methodology (equal), software (supporting), validation (equal), writing – original draft (equal), writing – review and editing (equal). **Suraj Baral:** conceptualization (equal), data curation (equal), formal analysis (lead), investigation (equal), methodology (lead), software (lead), supervision (equal), validation (lead), visualization (equal), writing – original draft (equal), writing – review and editing (equal). **Bhawana Pandey:** data curation (equal), methodology (supporting), writing – original draft (supporting). **Pratikshya Pathak:** data curation (equal), methodology (supporting), writing – original draft (supporting). **Bhupendra Kumar:** conceptualization (equal), funding acquisition (equal), supervision (equal), validation (equal), visualization (equal), writing – original draft (supporting), writing – review and editing (equal). **Pratap Karki:** validation (equal), visualization (equal), writing – review and editing (equal). **Sagun Narayan Joshi:** conceptualization (equal), project administration (equal), resources (equal), visualization (equal), writing – review and editing (equal). **Ananda Kumar Sharma:** project administration (equal), visualization (equal). **Madan Prasad Upadhya:** conceptualization (equal), validation (equal), writing – review and editing (equal).

## Conflicts of Interest

The authors declare no conflicts of interest.

## Supporting information


**Data S1:** ece372325‐sup‐0001‐FileS1.

## Data Availability

The data that support the findings of this study is available in the S1 of this article.
